# Supplementary material for: A Natural qE- and qZ-Deficient Alga Retains Functional Plant-Like and Chlorophycean Violaxanthin De-Epoxidases
Source: bioRxiv. 2026 Oct 2:2026.09.26.754548. Preprint. [Version 1] doi: 10.64898/2026.09.26.754548 (PMC13648494; doi:10.64898/2026.09.26.754548)
Supplement: Supplementary file 1 [file media-1.pdf]

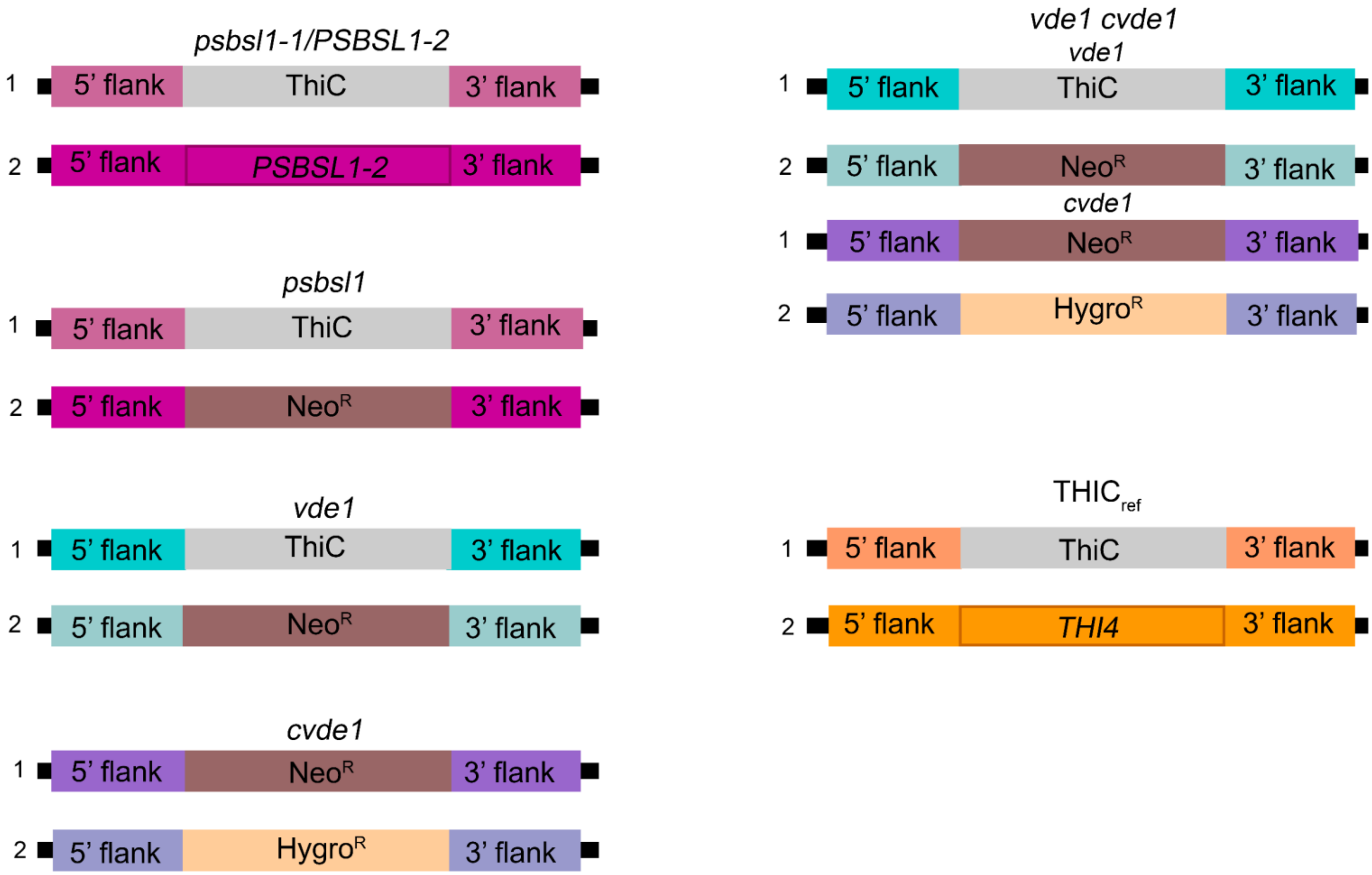

Supplementary Figure 1. Schematic summary of the target loci in the mutants generated in this study. Each allele was targeted with a construct containing a selection marker flanked by homologous arms on the 5’ and 3’ side. Fragments not to scale.
