# Supplementary material for: A Natural qE- and qZ-Deficient Alga Retains Functional Plant-Like and Chlorophycean Violaxanthin De-Epoxidases
Source: bioRxiv. 2026 Oct 2:2026.09.26.754548. Preprint. [Version 1] doi: 10.64898/2026.09.26.754548 (PMC13648494; doi:10.64898/2026.09.26.754548)
Supplement: Supplementary file 4 [file media-4.pdf]

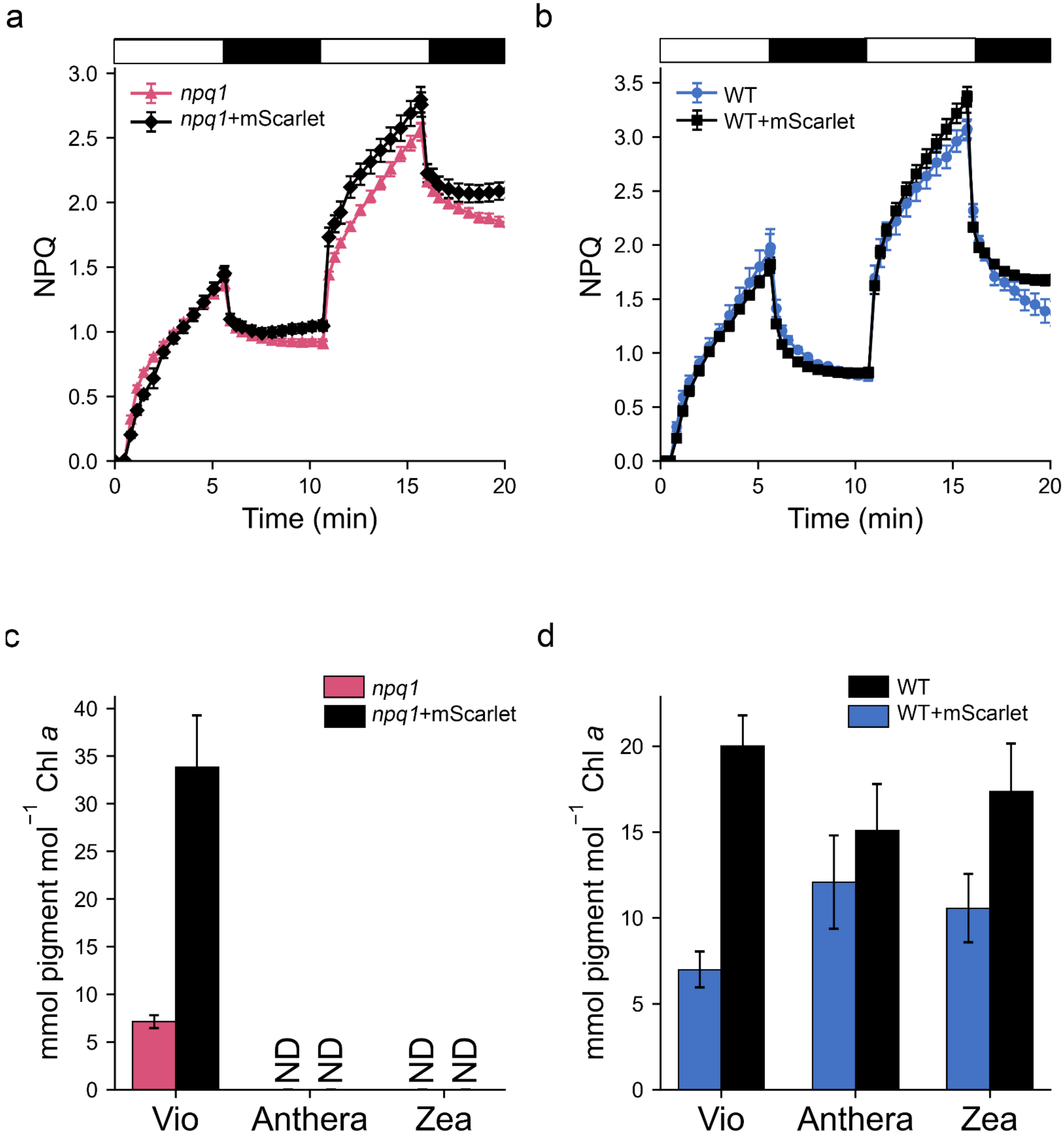

Supplementary Figure 4. mScarlet does not affect NPQ in the *npq1* or WT *Nicotiana*. NPQ of **a)** *npq1* versus *npq1* expressing mScarlet (*npq1* + mScarlet) and **b)** WT versus WT expressing mScarlet (WT + mScarlet). VAZ pigments in **c)** *npq1* versus *npq1* expressing mScarlet and **d)** WT versus WT expressing mScarlet. Pigments were extracted after 30 minutes of 1000 µmol photons m^{-2} s^{-1} actinic light. Values are average of replicate leaves and error bars represent standard error (n=6). ND, not detected.
